# The origin of a novel gene through overprinting in *Escherichia coli*

**DOI:** 10.1186/1471-2148-8-31

**Published:** 2008-01-28

**Authors:** Luis Delaye, Alexander DeLuna, Antonio Lazcano, Arturo Becerra

**Affiliations:** 1 Facultad de Ciencias, Universidad Nacional Autónoma de México. Apdo. Postal 70-407, Cd. Universitaria, 04510 México DF, México; 2 Department of Systems BiologyHarvard Medical School, 200 Longwood Ave, WAB 523 Boston, MA 02115, USA

## Abstract

**Background:**

Overlapped genes originate by a) loss of a stop codon among contiguous genes coded in different frames; b) shift to an upstream initiation codon of one of the contiguous genes; or c) by overprinting, whereby a novel open reading frame originates through point mutation inside an existing gene. Although overlapped genes are common in viruses, it is not clear whether overprinting has led to new genes in prokaryotes.

**Results:**

Here we report the origin of a new gene through overprinting in *Escherichia coli *K12. The *htgA *gene coding for a positive regulator of the sigma 32 heat shock promoter arose by point mutation in a 123/213 phase within an open reading frame (*yaaW*) of unknown function, most likely in the lineage leading to *E. coli *and *Shigella sp*. Further, we show that *yaaW *sequences coding for *htgA *genes have a slower evolutionary rate than those lacking an overlapped *htgA *gene.

**Conclusion:**

While overprinting has been shown to be rather frequent in the evolution of new genes in viruses, our results suggest that this mechanism has also contributed to the origin of a novel gene in a prokaryote. We propose the term *janolog *(from *Jano*, the two-faced Roman god) to describe the homology relationship that holds between two genes when one originated through overprinting of the other. One cannot dismiss the possibility that at least a small fraction of the large number of novel ORPhan genes detected in pan-genome and metagenomic studies arose by overprinting.

## Background

The origin of novelty is one of the central questions in evolutionary biology. Paralogy, orthology, and xenology, as well as domain shuffling between genes, account for a large part of the evolution of protein families at the sequence level [1]. All these mechanisms require the previous existence of coding sequences. Whether novel sequences in cells could arise from spontaneous point mutation remains an open question.

In some cases, two or more genes are known to be encoded in the same DNA region [2]. Overlapping genes have been known since the beginning of virus complete genome sequencing, as exemplified by the gene B of the single stranded DNA bacteriophage ϕX174, which is completely contained within gene A [3]. It has been suggested that overlapping genes originate by spontaneous point mutation due to a mechanism known as overprinting [4]. Overlapping genes could arise by: a) extension of one ORF into other by loss of a stop codon; b) shift to an upstream initiation codon in adjacent genes; or by c) generation of a totally new ORF inside a previous existing ORF by point mutation. The different ways in which two overlapping genes can be coded are known as phases [5], and the different phases determine, in turn, the evolutionary pressure that overlapping genes exert on each other [6,7].

In has been shown that overprinting is an important mechanism for the origin of new genes in viruses [4,8]. Overlapped genes are also a relative common feature of prokaryotic genomes. For instance, it has been suggested that in prokaryotes, where approximately 80% of the overlaps comprise less than 30 base pairs, the phase of coding and their distribution, among other characteristics of overlapping genes are most consistent with the hypothesis that overlaps participate in the regulation of gene expression [9]. A comparative study among the genomes of *Mycoplasma genitalium *and *Mycoplasma pneumoniae *found that most overlapped genes were generated primarily due to the loss of a stop codon, the absence of which resulted in elongation of the 3' end of the gene's coding region [10]. Overlapped genes can also be classified according to their direction of transcription into three categories, namely "convergent" (-> <-), "unidirectional" (-> ->) and "divergent" (<- ->). In a study that included the analysis of complete genome sequences from 50 bacterial species [11] it was shown that most overlapped genes are coded following the unidirectional pattern. This pattern was confirmed in a comparative study among nine bacteria [12]. Overlapped genes have also been found in mitochondria. For instance, a small protein [UniProtKB: Q6EMS7] of 66 amino acids (gene A6L) overlaps 40 bases into the gene coding for ATPase-6 in bovine mitochondria [13]. Because the formation of overlapping genes necessarily involves the evolution of a coding region from non-coding DNA or from a different frame in coding DNA, their study might help to understand *de novo *evolution of coding regions [5]. The pattern of natural selection among 71 pairs of 3' overlapping genes (all of them having an overlap larger than 15 nucleotides) conserved at least in two prokaryotic genomes showed a statistically significant bias toward the 123/132 phase, thus ensuring the least mutual constraint on non-conservative amino acid replacements in both overlapping coding sequences [5]. Here, we show that the mechanism of overprinting has also contributed to the origin of a new gene of identifiable function in *Escherichia coli*.

## Results

**Figure 1:**
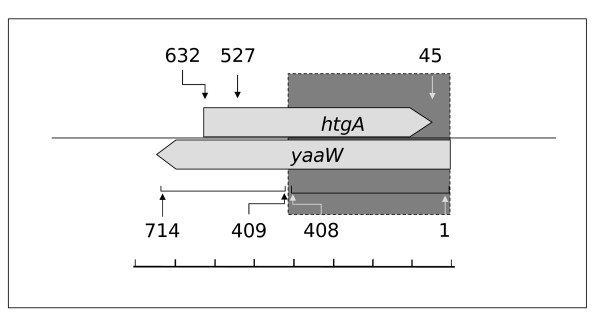
**Overlapped genes in *E.coli***. Overlapped *yaaW *[UniProtKB:P75617] and *htgA *[UniProtKB:P28697] genes in *E. coli*. Numbers indicate nucleotide residue for *yaaW *gene. The initiation codon of *hgtA *has been predicted to be in nucleotide 632 [14], and more recently in 527 [23]. A gray box indicates the region where we have detected a lowering in the rate of evolution of *yaaW *sequences with an *htgA *overlapped gene.

We have identified a possible case of evolution of a new gene and a new function by overprinting in *E. coli *K12. The overlapping genes are the gene for the positive regulator for sigma 32 heat shock promoter (*htgA*) [UniProtKB:P28697] and the gene *yaaW *[UniProtKB:P75617], whose hypothetical protein product has been classified as an Unidentified Protein Family 0174 in Pfam database [Pfam:UPF0174] (Figure 1). The genes are coded in the 123/213 phase (*htgA *in the (+) strand, and *yaaW *in the (-) strand), meaning that the third nucleotide of the (+) codon overlaps with the 3 nucleotide of the (-) codon. The overlap comprises the 591 nucleotides of the full *htgA *gene.

Although NCBI gene annotation tables provide coordinates for the position of genes in genomes, caution should be taken, since over-annotation and sequencing errors might mislead the identification of true genes. While there is *in vitro *experimental evidence for the existence of *htgA *[14], this does not hold true for *yaaW*. Therefore, we have searched for indirect evidence for its existence by asking whether there are: a) *yaaW *homologs in other genomes; b) domain fusions of homologs to the *yaaW *protein product with other protein domains; and c) detectable biases in the ratio of synonymous (*ps*) versus non-synonymous (*pn*) substitutions, i.e., departures from neutrality (*ps *≠ *pn*) among *yaaW *sequences that would suggest the action of natural selection.

### Phylogenetic distribution of *htgA *and *yaaW *genes

**Figure 2:**
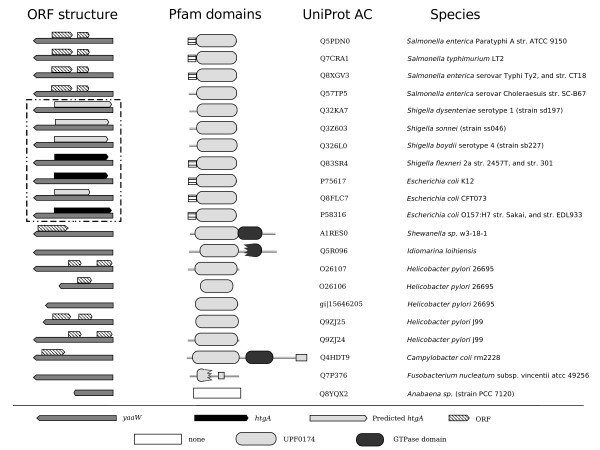
**Phylogenetic distribution of *yaaW *and *htgA *overlapped genes**. Phylogenetic distribution, open reading frames of overlapped genes, and protein domain organization of coded protein sequences according to Pfam database. ORF structure: Open reading frame of *yaaW *sequences is shown using left oriented dark-gray arrows; genome-annotated *htgA *sequences using right oriented black arrows; predicted *htgA *genes in this study right oriented pale-gray arrows; non-coding open reading frames right-oriented hatched arrows. Pfam domains: Pale-gray boxes indicated [Pfam:UPF0174] Pfam domain present in *yaaW *protein products; dark-gray boxes indicates GTPase domain of unknown function [Pfam:MMR_HSR1]. In the *yaaW *gene coding for [UniProtKB:Q8FLC7] the predicted *htgA *gene is shorter due to a non-sense mutation. Complete names of proteins are given in Additional file 3.

According to NCBI gene annotation tables, *htgA *is present in three *E. coli *and one *Shigella flexneri *strains. Furthermore, based on *i*) sequence similarity; *ii*) ORF prediction by ORF Finder; and *iii*) lowered rates of sequence evolution of *yaaW *sequences containing overlapped *htgA *genes (see below), we suggest that homologs to *htgA *are also present in all the other *E. coli *and *Shigella sp*. genomes analyzed here, adding up to a total of nine *htgA *sequences (Figure 2). On the other hand, *yaaW *has a wider phylogenetic distribution. This gene is present in 16 γ-proteobacteria and three ε-proteobacteria. *yaaW *can be identified as a highly divergent sequence in *Nostoc sp *PCC7120 a filamentous cyanobacteria and in a fusobacteria. In contrast with *htgA*, which is present as a single copy, some of the genomes analyzed here have paralogous copies of *yaaW *homologs. There are two genomes endowed with more than one copy of *yaaW*; *Helicobacter pylori *J99 has two copies, while *H. pylori *26695 has three copies. In total, we detected 24 homologs of *yaaW*. Since there are three pairs of identical sequences at the nucleotide level (each pair is coded on different strains from the same specie), there are only 21 UniProtKB codes (Figure 2). Multiple sequence alignment of *yaaW *protein coding genes is shown in Additional file 1. The wider phylogenetic distribution of *yaaW *suggests that *htgA *originated by overprinting in an ancestral copy of extant *yaaW *sequences (Complete names of proteins are given in Additional file 2).

We detected no homologs among viruses to the protein product of *htgA *in the non-redundant database. Besides a small similarity of the DnaC protein [UniProtKB:Q9AZV4] from *Lactococcus *phage bIL286 (41 amino acids identities along 152 residues, and BLAST e-value of 1.7) to the *yaaW *protein product. This appears to be a false positive since similarity was not confirmed by Pfam database. These negative results strongly suggest, but do not prove, that *yaaW *and *htgA *did not originate in a viral genome.

### Protein domain identification of *yaaW *and *htgA *coded proteins

The *yaaW *coded proteins match to [Pfam:UPF0174] profile in Pfam database, the only exception is the hypothetical protein alr3689 [UniProtKB:Q8YQX2] from *Nostoc *sp. PCC 7120 that exhibits a marginal HMMER E-value of 0.048, comprising 156 amino acids out of 185 (0.01 is considered significant in Pfam). However, a BLAST search of *E. coli yaaW *coded protein sequence [UniProtKB:P75617] to our database of proteins from complete genomes matches the cyanobacterial protein alr3689 sequence with a significant e-value of 2e-05 BLAST to a 86 amino acid stretch of the protein. The protein alr3689 seems to be a highly divergent homolog of *yaaW *having the UPF0174 domain. *yaaW *has fused to a GTPase domain [Pfam:MMR_HSR1] in *Shewanella *sp. and *Campylobacter coli*, which has a wide phylogenetic distribution but unknown function. *yaaW *is also fused to a fraction of this domain in the γ-proteobacteria *Idiomarina loihiensis *(Figure 2). This GTPase domain is fused in other proteins with a domain of unknown function: [Pfam:DUF933] related to the ubiquitin and to another GTPase domain [Pfam:GTP1_OBG] which have been shown to be important in normal cell metabolism in *Schizosaccharomyces pombe *[15]. The fusion of some *yaaW *homologs to a GTPase domain suggests that *yaaW *codes for a functional protein. On the other hand, there is no available Pfam domain for the *htgA *gene at the time being.

### Phylogenetic analysis

**Figure 3:**
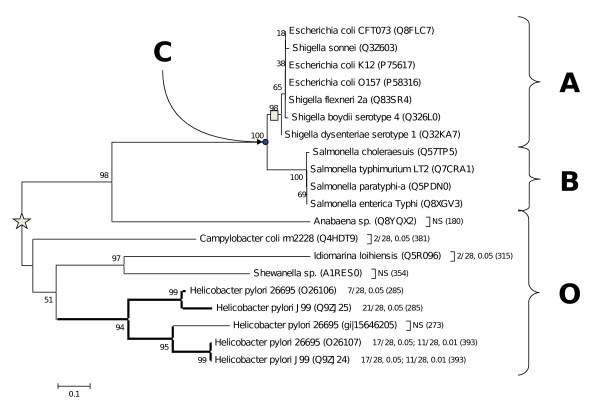
**Phylogenetic analysis of *yaaW *protein coded sequences**. Minimum Evolution tree of *yaaW *protein coding sequences (number in branches denotes 1000 bootstraps). A gray vertical bar indicates the branch where *htgA *overlapped gene hypothetically originated. A star indicates the placement of the root according to the midpoint root method. A, B and O denotes the groups of sequences used to detect the changes in rate evolution of *yaaW *sequences having and overlapped *htgA *gene. Node C is a hypothetical ancestral sequence. Darker branches indicate those sequences having the larger number of statistically significant relative rate tests when used as outgroups. Numbers following the names of proteins indicate the following: number of statistically significant comparisons/out of total number of comparisons, level of significance (number of amino acids involved in the relative rate taste). NS stands for: non-significant.

With the exception of the sequence from *Nostoc *sp. that branches among the proteobacteria, the phylogeny of *yaaW *coded proteins conforms approximately to canonical rRNA-based tree (Figure 3).

### Synonymous versus non-synonymous substitutions

**Figure 4:**
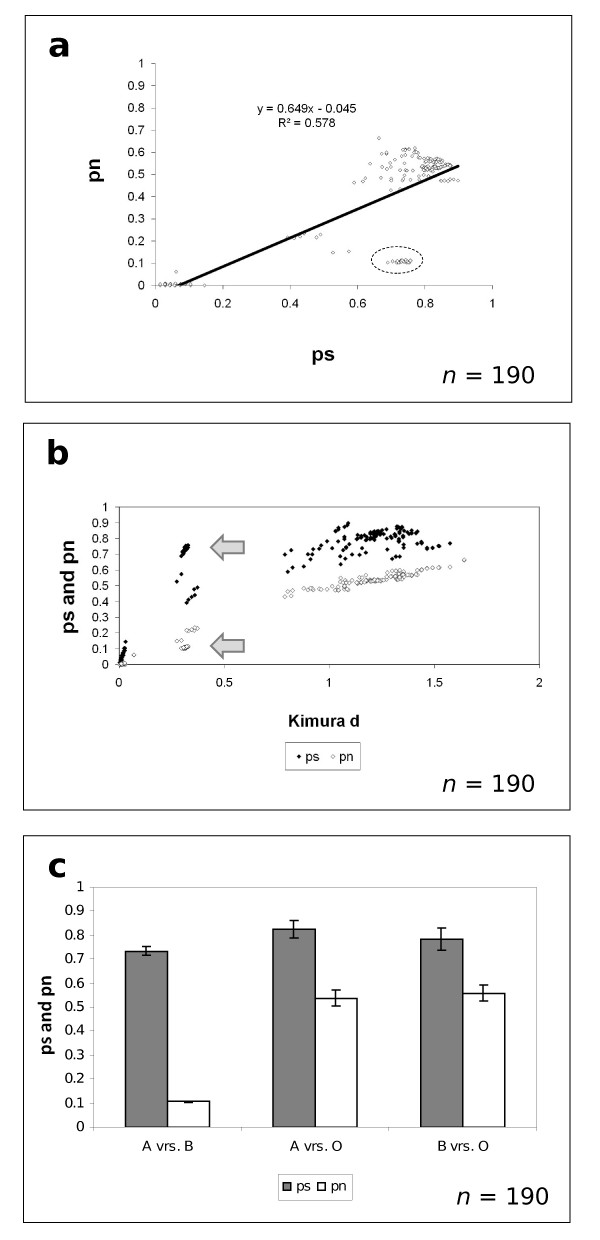
**Synonymous versus non-synonymous substitutions**. a) Proportion of synonymous (*pn*) versus non-synonymous (*ps*) substitutions for *yaaW *sequences. The dotted oval indicate comparisons between group A and B sequences (y = 0.649x - 0.045; r^2 ^= 0.578); b) Distribution of *ps *(black dots) and *pn *(white dots) against Kimura distance for *yaaW *genes. Arrows indicate comparisons between group A and B sequences; c) Number of *pn *versus *ps *values of *yaaW *sequences between groups (A, B and O), bars indicate standard deviation.

The relationship between synonymous (*ps*) versus non-synonymous (*pn*) substitutions among all pairs of *yaaW *sequences is shown in Figure 4a. The dashed oval indicates comparisons between group A and B sequences (as defined in Figure 3). In figure 4b, the values of *pn *and *ps *are plotted against Kimura distances for each pair of *yaaW *sequences. As shown, the excess of synonymous versus non-synonymous substitutions is suggestive of purifying selection. This excess is especially important between sequences belonging to group A and group B (Figure 4c), indicating a higher degree of restriction to non-synonymous changes imposed by *htgA *on *yaaW *sequences. Purifying selection is statistically significant for all *yaaW *sequences (Table 1).

**Table 1:** Natural selection analysis. The mean of *p *values for all the comparisons among all *yaaW *and *htgA *homologs plus standard deviations, SD. * *p *< 0.05; ** *p *< 0.01. Neutrality: a statistically significant *p *value indicates non-neutral evolution; Purifying selection: a statistically significant *p *value indicates purifying selection

	Neutrality	Purifying selection
*yaaW *(all sequences)	0.042 ± 0.1215 *	0.024 ± 0.0869 *
A versus B	2.079E-09 ± 1.6218E-09 **	1.168E-09 ± 6.5436E-10 **
A versus O	0.009 ± 0.0266 **	0.005 ± 0.0139 **
B versus O	0.031 ± 0.0696 *	0.015 ± 0.0337 *

*htgA *(all sequences)	0.116 ± 0.2171	0.068 ± 0.1456
A versus B	6.69E-06 ± 8.3638E-06 **	2.378E-06 ± 3.0695E-06**
A versus O	0.118 ± 0.2358	0.057 ± 0.1187
B versus O	0.097 ± 0.2122	0.050 ± 0.1050

The pattern of synonymous and non-synonymous substitutions among *htgA *sequences also suggests purifying selection (Table 1). However this pattern could be the reflection of the accumulation of purifying mutations among *yaaW *genes and the 123/213 phase of the overlap. Therefore, synonymous and non-synonymous mutations in *yaaW *will affect the similarly to *htgA*. This is an unexpected pattern for a novel sequence since it has been shown that new genes originating through overprinting in viruses show an excess of non-synonymous substitutions, indicating the action of positive natural selection [8].

### Lower rate of evolution of *yaaW *sequences with overlapped *htgA *genes

DNA sequences coding for overlapped genes are expected to evolve at a lower rate than those DNA sequences coding for only one gene [6,7]. Accordingly, we have analyzed the substitution rates of closely related *yaaW *genes with and without the overlapped *htgA *using a non-parametric relative rate test [16] for all combinations of three sequences consisting of an out-group (O) sequence, and two in-group (A and B) sequences (see methods and Figure 3).

**Figure 5:**
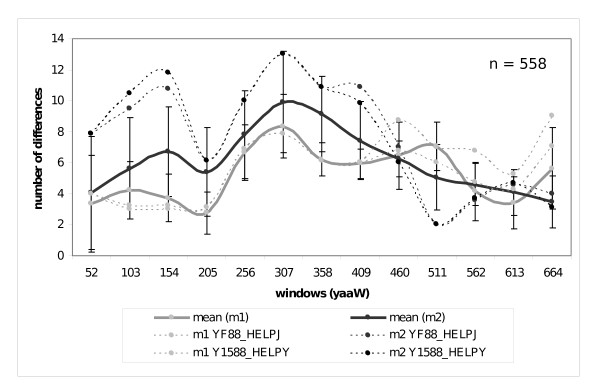
**Differences in rate substitution among *yaaW *sequences**. Number of mutations exclusive of group A sequences (m1 mutations) versus number of exclusive mutations of group B sequences (m2 mutations) in *yaaW *genes in windows of 102 nucleotides. X values denotes the mid position of each window in *E. coli yaaW *sequence. Contiguous windows overlap 51 nucleotides. Thick lines denote the mean number of exclusive mutations for m1 and m2 changes. Dashed lines indicated the number of m1 and m2 changes for sequences showing the most extreme values. Each point is accompanied by its standard deviation.

**Figure 6:**
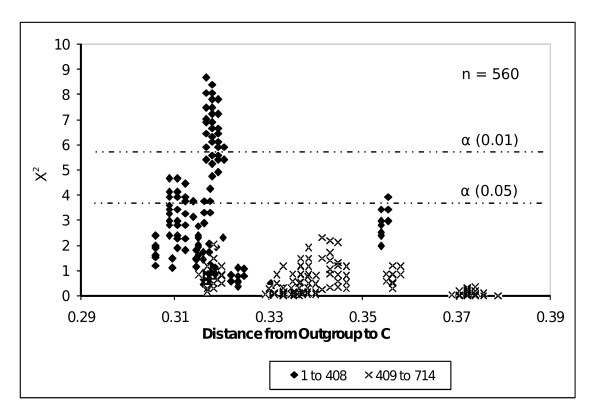
**Statistic analysis**. Distribution of Chi-square values of relative rate tests against distance of out-group sequence (O) to node C. Black dots correspond to the first 408 nucleotides of *yaaW *and crosses correspond to the rest of the gene. The 0.005 and 0.001 significance levels are indicated with dotted lines.

In-group *yaaW *sequences lacking the overlap (B sequences in Figure 3) have accumulated more exclusive mutations (m2 changes in Figure 5) in the first 409 nucleotides than those in-group *yaaW *genes endowed with the overlap (A sequences in Figure 3 and m1 changes in Figure 5). This suggests that *htgA *exerts an evolutionary pressure to *yaaW *in their first 409 nucleotides. Accordingly, we have subdivided the *yaaW *alignment in two sections. The first one comprises nucleotides 1 to 408, while the second one includes nucleotides 409 to 714. We have then applied the Tajima test [16] to both sections independently. As seen in Figure 6, many of the differences are significant at α = 0.05 for the first 408 nucleotides, and for some comparisons even at the α = 0.01 level. This is particularly true for the genes encoding for [UniProtKB: O26107] and [UniProtKB: Q9ZJ24] protein sequences (they also align best with A and B sequences). However, not all comparisons give statistically significant results. It is likely that signal erosion in sequences having experienced more substitutions may explain in part lack of statistically significant results in some relative rate tests, since there seems to be a tendency of lower Chi-square values towards increasing genetic distance (Figure 6).

## Discussion

Ever since Darwin, homology in biology has been explained by common descent. With the advent of the molecular structure of nucleic acids and proteins, the concept of homology was further refined to describe more precisely the phylogenetic relationships among genes (reviewed in [17]). Accordingly, genes separated by speciation events are referred as *orthologs*, genes duplicated inside the same genome are named as *paralogs*, and horizontally transferred genes are known as *xenologs*. Further on, in symbiogenesis lineages of homologous genes previously separated by lineage divergence, became associated in a single cellular system. The term *synology *has been proposed to describe the phylogenetic relationship among such genes [18]. However, no terminology has been employed to describe the phylogenetic relationship between homologous overlapping genes. We suggest the term *janology *(from the two-faced Roman deity *Jano*) for the homology relationship among the sequences sharing the same DNA.

Other cases of novel genes have been described (i.e., *Jingwey*, [19]; *Sphinx*, [20];*Morpheus *[21]) and the origin of novel sequences through overprinting in viruses is well documented [4,8]. However, to the best of our knowledge, this is the first time that a novel gene originated through overprinting has been carefully described in a cell.

Caution should be taken since the existence of the *htgA *gene remains controversial. Although the gene has been cloned and its product originally characterized as a positive regulator of sigma 32 heat shock promoter [14], a recent study shows that this gene is not induced by sigma 32 as previously suggested [22]. Moreover, the initiation codon of *htgA *have recently being changed [23] and one of the predicted copies of *htgA *(Figure 2) has a smaller size due to a non-sense mutation. Given that there has not been more experimental work carried on *htgA*, we rely on indirect evidence of its existence based on the statistically significant lowering rate of evolution of those sequences putatively coding for *htgA*. It is worth mentioning that available evidence for the role of *htgA *included cloning of the *htgA *gene, RNA isolation and Northern (RNA) blots, protein labeling, and promoter mapping experiments [14]. One possibility for the lack of sigma 32 induction is that, as suggested by [22], *htgA *either requires additional regulators or is not recognized by sigma 32. Our study should stimulate more research on this subject in order to elucidate the true nature of *htgA*.

There is no available experimental evidence of the existence of the protein product of *yaaW*. Nevertheless, *i*) the existence of homolog sequences to *yaaW *in different genomes; *ii*) the fusion of *yaaW *sequences to other protein domains (a GTPase domain in this case); and *iii*) the higher ratio of *ps *versus *pn *substitutions, strongly suggest that *yaaW *truly codes for a functional polypeptide. Here we propose that *htgA *originated by overprinting (*de novo *origination of a new gene from a non-coding frame in DNA in a prokaryotic genome) at some moment in the clade leading to *E. coli *and *Shigella flexneri *species.

Recently, a survey of genetic diversity on the Sargasso Sea using shot-gun technology showed the existence of large number of previously undetected new genes [24]. Using a different approach, [25] have shown the existence of an open pan-genome for the species *Streptococcus agalactiae*. This indicates that the genome of *S. agalactiae *is constantly exchanging genes with other organisms in its environment and that novel sequenced genomes from this species will exhibit an approximate of 30 new genes not previously found in the other strains. In a recent re-annotation of *E. coli *genome [23], it was shown that despite much effort in understanding the functions of each gene, there still a number of uncharacterized genes unique to the genome of *E. coli *(the gene products of unknown function are divided into those containing a conserved domain (N = 145, 3.3%), those with (N = 233, 5.3%) or without (N = 238, 5.3%) a detectable homolog in the sequence databases). All these approaches suggest the existence of a large number of as yet uncharacterized new genes in the biosphere. This opens the possibility that some of such novel genes arose through point mutation from coding (overprinting) as well as non-coding sequences. It also suggests that the origin of the *novo *sequences is an ongoing process in prokaryotic evolution.

The number of gene and protein families in the biosphere continues to be an open area of research. For instance, the 1.69 release of SCOP database has 1536 superfamilies [26], and this is certainly an underestimate. The origin of each one of these families continues to be a matter of debate and several mechanisms may be involved. Overprinting is a clear example of how new protein families can originate independently from non-coding sequences. If the new gene (or the new region of the gene resulting from the loss of a stop codon) is in a different strand or in a different coding frame than the other overlapping gene, a new coding sequence is being generated *de novo*. If this new coding region is transcribed and translated and the product is able to fold properly, natural selection could recruit this protein for some function, and if successful, it can be fixed in the population, and may in the long term represent the origin of a new protein/domain family.

## Conclusion

We have described the origin of a novel gene of known function through overprinting in a prokaryote. Due to the wider phylogenetic distribution of *yaaW*, parsimony favors the origin of *htgA *by overprinting inside an already existing *yaaW *gene. The evidence of *htgA *being a functional gene is experimental [14], while the evidence for *yaaW *comes from: *i*) the existence of homolog sequences to *yaaW *in different genomes; *ii*) the fusion of *yaaW *sequences to other protein domains (a GTPase domain in this case); and *iii*) and the higher ratio of *ps *versus *pn *substitutions among *yaaW *sequences. Intriguingly there is no evidence of the action of positive natural selection among *htgA *homologs. This can be explained by a high degree of restriction imposed by *yaaW *due to the 123/213 phase of the overlap. The lower rate of evolution of *yaaW *sequences having an overlapped *htgA *gene is explained by the higher constraints of a DNA sequence coding for two genes. If our interpretation of the above evidence is correct, it is likely that the origin of *de novo *sequences from non-coding DNA in prokaryotes is an ongoing process.

## Methods

We have searched for all strict pairs of completely overlapped genes in available *E.coli *complete genomes. We have found 227 pairs of such genes (Additional file 3). After the exclusion of all overlapped genes annotated as: a) hypothetical; b) related to phages; c) lack of homologs to one (or both) of the overlapped genes, there are 7 pairs left. Among these seven pairs, there is experimental evidence for the existence of the hypothetically newer gene (the one with fewer homologs) only the *htgA*-*yaaW *pairs (three pairs). Among the four pairs left, the pair conformed by the molecular chaperone DnaK (GI identification number: 26245936; 500 homologs found) and the putative glutamate dehydrogenase (GI identification number: 26245935; 6 homologs found) looks like a promising alternative. However we have filed to detect a difference in the rate of evolution with the method used here. We have left all the previous information available through the Additional file 3 for further studies. Therefore, we have selected the *htgA*-*yaaW *pair based on the experimental evidence of the existence of the gene with fewer homologs (*htgA*) and the presence of homologs to the other gene (*yaaW*) in genomes belonging to different bacterial species.

Based on the recently re-annotated gene table of *E. coli *K12 genome [23] these genes have the following coordinates, *yaaW *[UniProtKB:P75617] and *htgA *[UniProtKB:P28697] (*yaaW*: locustag K-12 ECK0011, left nucleotide 10643, right nucleotide 11356, direction of transcription (-); *htgA*: locustag K-12 ECK0012, left nucleotide 10830, right nucleotide 11315, direction of transcription (+)).

We have searched for all homologs of the protein products of both genes in a database of 416 prokaryotic chromosomes (including main chromosomes and accessory genetic elements like plasmids) from 251 prokaryotic organisms using BLAST [27] searches (e value cut-off 0.001) as well as in the NCBI non-redundant database for virus homologs.

Protein domain identification of homologs thus identified was assigned according Pfam database [28]. Open reading frames of *htgA *genes potentially coded in *yaaW *sequences were predicted using ORF Finder at NCBI [29]. Genomic coordinates of each of the genes were searched in NCBI gene annotation tables [30].

### Multiple alignment

Multiple sequence alignment of homologs to *yaaW *protein products was performed using ClustalW [31] as implemented in Bioedit [32] and edited manually following the alignment of protein family [Pfam:UPF0174] deposited in Pfam. Conserved residues were identified using Jalview [33]. DNA sequences of *yaaW *genes were aligned following [Pfam:UPF0174] protein sequence alignment, in order to preserve codon positions in the multiple DNA sequence alignment.

### Phylogenetic analysis

A Minimum-Evolution tree of amino acid sequences homologs to *yaaW *protein product was inferred using Poisson correction, constant rate homogeneity and 100 bootstrap replications, using MEGA3.1 software [34].

### Synonymous and non-synonymous differences

The proportion of synonymous (*ps*) versus non-synonymous (*pn*) differences were calculated following Nei-Gojobori method [35] as implemented in MEGA3.1 software [34].

### Selection test

In order to understand the pattern of selection (purifying selection, neutrality or positive selection) we have applied a large sample *Z *test as implemented in MEGA3.1 software [34] to all pairs of *yaaW *genes, as well as to all pairs of *htgA *homologs.

### Relative rate test

A non-parametric relative rate test following Tajima [16] was applied to all combinations of three sequences consisting of an out-group (O) sequence, and two in-group (A and B) sequences (see Figure 3 to identify sequences belonging to each group) using a Perl script RRT.pl available upon request. The analysis was performed using DNA sequences. The method counts the number of substitutions exclusive of A sequences (m1 substitutions) against the number of substitutions exclusive of the B sequences (m2 substitutions), using an out-group sequence O as a reference to identify such changes. The statistical significance is evaluated using a Chi-square test. We have also analyzed if there are rate differences across the overlap, therefore we have subdivided the multiple alignment of *yaaW *homologs in 13 windows of 102 nucleotides each (adjacent windows overlap by 51 nucleotides).

## Authors' contributions

LD conceived the study, participated in its design, conducted the sequence analysis and wrote the manuscript. ADL participated in the design of the study, the analysis of the results and helped to draft the manuscript. AL participated in the analysis of the results and made substantial improvements to the manuscript. AB participated in the design and coordination of the study, and helped to draft the manuscript. All authors read and approved the final manuscript.

## Supplementary Material

Additional File 1 **Sequence alignment**. Multiple alignment of protein coding sequences of *yaaW *homologs. Click here for file

Additional File 2 **Complete names of *yaaW *homologs**. Complete names of all proteins belonging to [Pfam:UPF0174]. Click here for file

Additional File 3 **Overlapping sequences in *E. coli *genomes**. Analysis of all strict pairs of completely overlapped sequences available in *E. coli *genomes. Click here for file

## References

[B1] Henikoff S Greene EA Pietrokovski S Bork P Attwood TK Hood L Gene families: the taxonomy of protein paralogs and chimeras Science 1997 278 609 614 10.1126/science.278.5338.609 9381171

[B2] Li W-H Molecular Evolution 1997 Sinauer Associates

[B3] Sanger F Coulson AR Friedmann T Air GM Barrell BG Brown NL Fiddes JC Hutchison CA III Slocombe PM Smith M The nucleotide sequence of bacteriophage ϕX174 J Mol Biol 1978 125 225 246 10.1016/0022-2836(78)90346-7 731693

[B4] Keese PK Gibbs A Origins of genes: "big bang" or continuous creation? Proc Natl Acad Sci USA 1992 89 9489 9493 10.1073/pnas.89.20.9489 1329098 PMC50157

[B5] Rogozin IB Spiridonov AN Sorokin AV Wolf YI Jordan IK Tatusov RL Koonin EV Purifying and directional selection in overlapping prokaryotic genes Trends Genet 2002 18 228 232 10.1016/S0168-9525(02)02649-5 12047938

[B6] Miyata T Yasunaga T Evolution of overlapping genes Nature 1978 272 532 535 10.1038/272532a0 692657

[B7] Krakauer DC Stability and evolution of overlapping genes Evolution 2000 54 731 739 10937248 10.1111/j.0014-3820.2000.tb00075.x

[B8] Pavesi A Origin and evolution of overlapping genes in the family Microviridae J Gen Virol 2006 87 1013 1017 10.1099/vir.0.81375-0 16528052

[B9] Johnson ZI Chisholm SW Properties of overlapping genes are conserved across microbial genomes Genome Res 2004 14 2268 2272 10.1101/gr.2433104 15520290 PMC525685

[B10] Fukuda Y Washio T Tomita M Comparative study of overlapping genes in the genomes of *Mycoplasma genitalium *and *Mycomplasma pneumoniae* Nucleic Acids Res 1999 27 1847 1853 10.1093/nar/27.8.1847 10101192 PMC148392

[B11] Fukuda Y Nakayama Y Tomita M On dynamics of overlapping genes in bacterial genomes Gene 2003 323 181 187 10.1016/j.gene.2003.09.021 14659892

[B12] Sakharkar KR Sakharkar MK Verma Ch Chow TTK Comparative study of overlapping genes in bacteria, with special reference to *Rickettsia prowazekii *and *Rickettsia conorii* Int J Syst Evol Microbiol 2005 55 1205 1209 10.1099/ijs.0.63446-0 15879256

[B13] Fearnley IM Walker JE Two overlapping genes in bovine mitochondrial DNA encode membrane components of ATP synthase EMBO J 1986 5 2003 2008 2875870 10.1002/j.1460-2075.1986.tb04456.x PMC1167070

[B14] Missiakas D Georgopoulos C Raina S The *Escherichia coli *Heat Shock Gene *htpY*: Mutational Analysis, Cloning, Sequencing, and Transcriptional Regulation J Bacteriol 1993 175 2613 2624 8478327 10.1128/jb.175.9.2613-2624.1993 PMC204563

[B15] Hudson JD Young PG Sequence of the *Schizosaccharomyces pombe *gtp1 gene and identification of a novel family of putative GTP-binding proteins Gene 1993 125 191 193 10.1016/0378-1119(93)90327-Y 8462872

[B16] Tajima F Simple methods for testing molecular clock hypothesis Genetics 1993 135 599 607 8244016 10.1093/genetics/135.2.599 PMC1205659

[B17] Fitch WM Homology a personal view on some of the problems Trends Genet 2000 16 227 231 10.1016/S0168-9525(00)02005-9 10782117

[B18] Gogarten JP Which is the Most Conserved Group of Proteins? Homology-Orthology, Paralogy, Xenology, and the Fusion of Independent Lineages J Mol Evol 1994 39 541 543 10.1007/BF00173425 7807544

[B19] Long M Langley CH Natural selection and the origin of *jingwei*, a chimeric processed functional gene in *Drosophila* Science 1993 260 91 95 10.1126/science.7682012 7682012

[B20] Wang W Brunet FG Nevo E Long M Origin of *sphinx*, a young chimeric RNA in *Drosophila melanogaster* Proc Natl Acad Sci USA 2002 99 4448 4453 10.1073/pnas.072066399 11904380 PMC123668

[B21] Johnson ME Viggiano L Bailey JA Abdul-Rauf M Goodwin G Rocci M Eichler EE Positive selection of a gene family during the emergence of humans and African apes Nature 1994 413 514 519 10.1038/35097067 11586358

[B22] Nonaka G Blankschien M Herman C Gross CA Rhodius VA Regulon and promoter analysis of the *E. coli *heat-shock factor, sigma32, reveals a multifaceted cellular response to heat stress Genes Dev 2006 20 1776 1789 10.1101/gad.1428206 16818608 PMC1522074

[B23] Riley M Abe T Arnaud MB Berlyn MK Blattner FR Chaudhuri RR Glasner JD Horiuchi T Keseler IM Kosuge T Mori H Perna NT Plunkett G Rudd KE Serres MH Thomas GH Thomson NR Wishart D Wanner BL *Escherichia coli *K-12: a cooperatively developed annotation snapshot–2005 Nucleic Acids Res 2006 34 1 9 10.1093/nar/gkj405 16397293 PMC1325200

[B24] Venter JC Remington K Heidelberg JF Halpern AL Rusch D Eisen JA Wu D Paulsen I Nelson KE Nelson W Fouts DE Levy S Knap AH Lomas MW Nealson K White O Peterson J Hoffman J Parsons R Baden-Tillson H Pfannkoch C Rogers YH Smith HO Environmental genome shotgun sequencing of the Sargasso Sea Science 2004 304 66 74 10.1126/science.1093857 15001713

[B25] Tettelin H Masignani V Cieslewicz MJ Donati C Medini D Ward NL Angiuoli SV Crabtree J Jones AL Durkin AS Deboy RT Davidsen TM Mora M Scarselli M Margarit y Ros I Peterson JD Hauser CR Sundaram JP Nelson WC Madupu R Brinkac LM Dodson RJ Rosovitz MJ Sullivan SA Daugherty SC Haft DH Selengut J Gwinn ML Zhou L Zafar N Khouri H Radune D Dimitrov G Watkins K O'Connor KJ Smith S Utterback TR White O Rubens CE Grandi G Madoff LC Kasper DL Telford JL Wessels MR Rappuoli R Fraser CM Genome analysis of multiple pathogenic isolates of *Streptococcus agalactiae*: implications for the microbial "pan-genome" Proc Natl Acad Sci USA 2005 102 13950 13955 10.1073/pnas.0506758102 16172379 PMC1216834

[B26] Andreeva A Howorth D Brenner SE Hubbard TJP Chothia C Murzin AG SCOP database in 2004: refinements integrate structure and sequence family data Nucleic Acid Res 2004 32 D226 D229 10.1093/nar/gkh039 14681400 PMC308773

[B27] Altschul SF Madden TL Schäffer AA Zhang J Zhang Z Miller W Lipman DJ Gapped BLAST and PSI-BLAST: a new generation of protein database search programs Nucleic Acids Res 1997 25 3389 3402 10.1093/nar/25.17.3389 9254694 PMC146917

[B28] Finn RD Mistry J Schuster-Böckler B Griffiths-Jones S Hollich V Lassmann T Moxon S Marshall M Khanna A Durbin R Eddy SR Sonnhammer ELL Bateman A Pfam: clans, web tools and services Nucleic Acids Res Database Issue 2006 34 D247 D251 10.1093/nar/gkj149 PMC1347511 16381856

[B29] ORF Finder (Open Reading Frame Finder) http://www.ncbi.nlm.nih.gov/

[B30] Entrez Genome Project http://www.ncbi.nlm.nih.gov/genomes/lproks.cgi

[B31] Thompson JD Higgins DG Gibson TJ CLUSTAL W: improving the sensitivity of progressive multiple sequence alignment through sequence weighting, position specific gap penalties and weight matrix choice Nucleic Acids Res 1994 22 4673 4680 10.1093/nar/22.22.4673 7984417 PMC308517

[B32] Hall TA BioEdit: a user-friendly biological sequence alignment editor and analysis program for Windows 95/98/NT Nucl Acids Symp Ser 1999 41 95 98

[B33] Clamp M Cuff J Searle SM Barton GJ The Jalview Aligment aEditor Bioinformatics 2004 12 426 427 10.1093/bioinformatics/btg430 14960472

[B34] Kumar S Tamura K Nei M MEGA3: Integrated software for Molecular Evolutionary Genetics Analysis and sequence alignment Brief Bioinform 2004 5 150 163 10.1093/bib/5.2.150 15260895

[B35] Nei M Gojobori T Simple methods for estimating the number of synonymous and nonsynonymous nucleotide substitutions Mol Biol Evol 1986 3 418 426 3444411 10.1093/oxfordjournals.molbev.a040410

