# Supplementary figures and images for: The origin of a novel gene through overprinting in Escherichia coli
Source: BMC Evol Biol. 2008 Jan 28;8:31. doi: 10.1186/1471-2148-8-31 (PMC2268670; doi:10.1186/1471-2148-8-31)

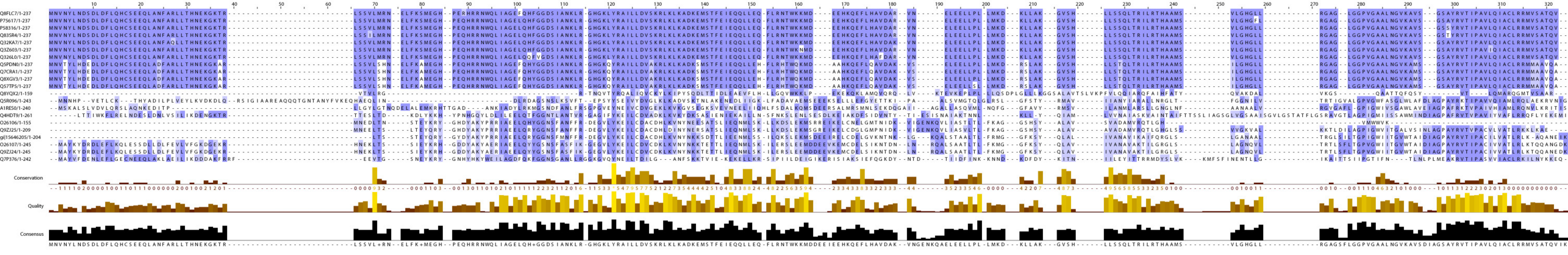

Supplement: Additional File 1 — Sequence alignment. Multiple alignment of protein coding sequences of yaaW homologs. [file 1471-2148-8-31-S1.PDF]
