# Supplementary material for: The origin of a novel gene through overprinting in Escherichia coli
Source: BMC Evol Biol. 2008 Jan 28;8:31. doi: 10.1186/1471-2148-8-31 (PMC2268670; doi:10.1186/1471-2148-8-31)
Supplement: Additional File 2 — Complete names of yaaW homologs. Complete names of all proteins belonging to [Pfam:UPF0174]. [file 1471-2148-8-31-S2.PDF]

Additional file 2

Complete names of all proteins belonging to [Pfam:UPF0174].

UniProt AC	Function	Species
Q5PDN0	hypothetical protein	*Salmonella enterica* subsp. *enterica* serovar Paratyphi A str. ATCC 9150
Q7CRA1	positive regulator	*Salmonella typhimurium* LT2
Q8XGV3	hypothetical protein	*Salmonella enterica* subsp. enterica serovar Typhi Ty2<br>*Salmonella enterica* subsp. enterica serovar Typhi str. CT18
Q57TP5	positive regulator for sigma H promoters	*Salmonella enterica* serovar Choleraesuis str. SC-B67
Q32KA7	putative oxidoreductase	*Shigella dysenteriae* serotype 1 (strain sd197)
Q3Z603	putative oxidoreductase	*Shigella sonnei* (strain ss046)
Q326L0	putative oxidoreductase	*Shigella boydii* serotype 4 (strain sb227)
Q83SR4	putative oxidoreductase	*Shigella flexneri* 2a str. 301<br>*Shigella flexneri* 2a str. 2457T
P75617	hypothetical protein	*Escherichia coli* K12
Q8FLC7	hypothetical protein yaaW	*Escherichia coli* CFT073
P58316	putative oxidoreductase	*Escherichia coli* O157:H7<br>*Escherichia coli* O157:H7 EDL933
A1RES0	small GTP-binding protein domain	*Shewanella* sp. w3-18-1
Q5R096	predicted C-terminal GTPase domain	*Idiomarina loihiensis*
O26107	hypothetical protein	*Helicobacter pylori* 26695
O26106	hypothetical protein	*Helicobacter pylori* 26695
gi|15646205	hypothetical protein	*Helicobacter pylori* 26695
Q9ZJ25	hypothetical protein	*Helicobacter pylori* J99
Q9ZJ24	hypothetical protein	*Helicobacter pylori* J99
Q4HDT9	hypothetical protein	*Campylobacter coli* rm2228
Q7P376	hypothetical protein	*Fusobacterium nucleatum* subsp. vincentii atcc 49256
Q8YQX2	hypothetical protein alr3689	*Nostoc sp.* PCC 7120

Table1. Sequences belonging to the UPF0174 protein family.
