# Supplementary material for: The origin of a novel gene through overprinting in Escherichia coli
Source: BMC Evol Biol. 2008 Jan 28;8:31. doi: 10.1186/1471-2148-8-31 (PMC2268670; doi:10.1186/1471-2148-8-31)
Supplement: Additional File 3 — Overlapping sequences in E. coli genomes. Analysis of all strict pairs of completely overlapped sequences available in E. coli genomes. [file 1471-2148-8-31-S3.DOC]

Additional file 3

| **NCBI code** | **Genetic element** | **Species** |
| --- | --- | --- |
| **NC_000913** | **chromosome** | ***Escherichia coli* K12 Genome ID:115** |
| **NC_007946** | **chromosome** | ***Escherichia coli* UTI89 Genome ID:19365** |
| **NC_007941** | **plasmid** | ***Escherichia coli* UTI89 Genome ID:19360** |
| **AC_000091** | **chromosome** | ***Escherichia coli* W3110 Genome ID:123455** |
| **NC_002695** | **chromosome** | ***Escherichia coli* O157:H7 str. Sakai Genome ID:176** |
| NC_002128 | plasmid | *Escherichia coli* O157:H7 str. Sakai Genoma_ID:15229 |
| NC_002127 | plasmid | *Escherichia coli* O157:H7 str. Sakai Genoma_ID:15228 |
| **NC_002655** | **chromosome** | ***Escherichia coli* O157:H7 EDL933 Genome ID:169** |
| NC_007414 | plasmid | *Escherichia coli* O157:H7 EDL933 Genoma_ID:18764 |
| **NC_004431** | **chromosome** | ***Escherichia coli* CFT073 Genome ID:267** |
| NC_008253 | chromosome | *Escherichia coli* 536 |
| NC_008563 | chromosome | *Escherichia coli* APEC O1 |

**Table 1**. *Escherichia coli* genomes scanned for pairs of overlapped genes. Chromosomes having strict pairs of completely overlapped sequences are shown in bold.


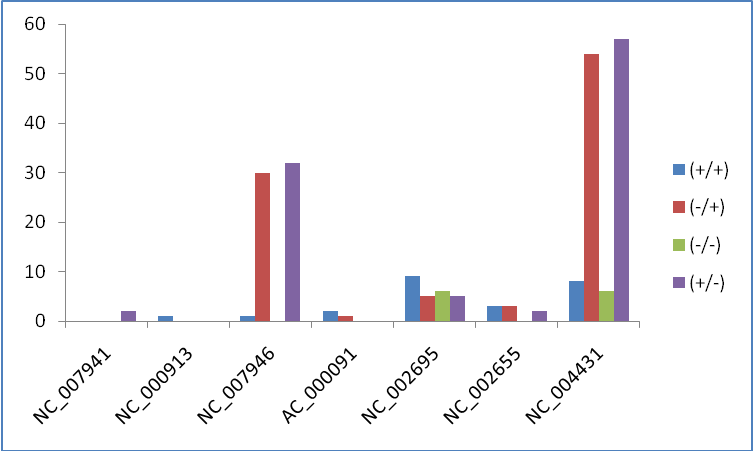


**Figure 1**. Distribution of pairs of overlapped genes among *Escherichia coli* genomes in four different coding phases.


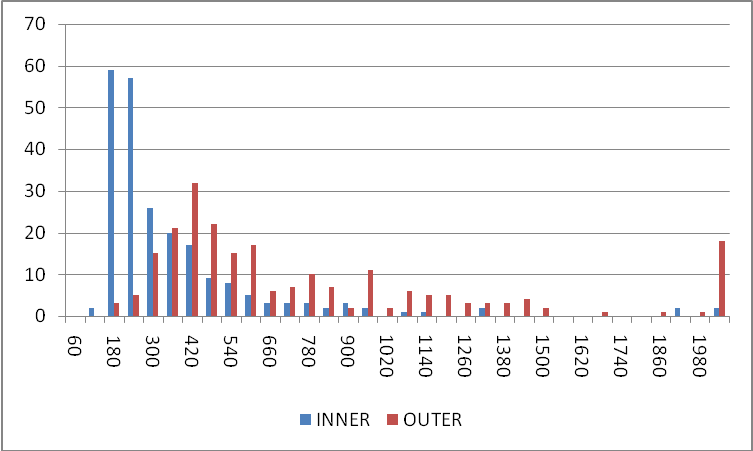


**Figure 2**. Size distribution of pairs of overlapped genes. INNER (genes coded inside other gene); OUTER (genes coded outside other genes).

**
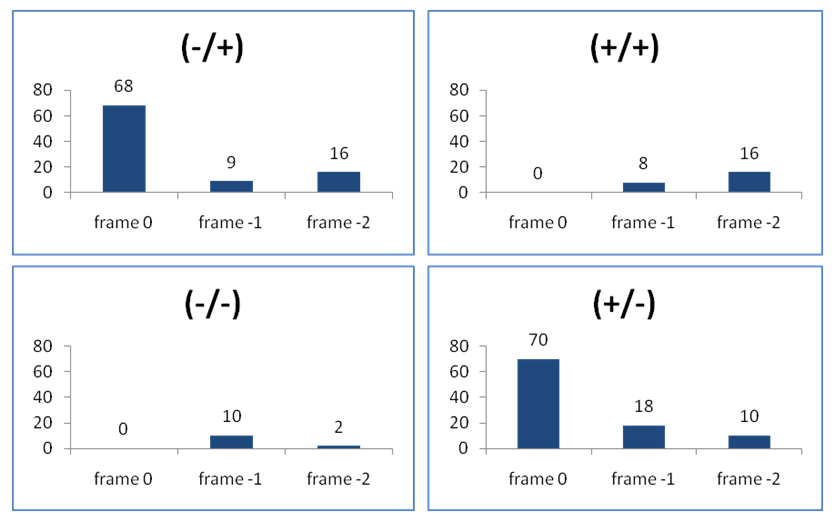
**

**Figure 3**. Distribution of phases among 227 pairs of overlapped genes in 6 *Escherichia coli* genomes and one plasmid.


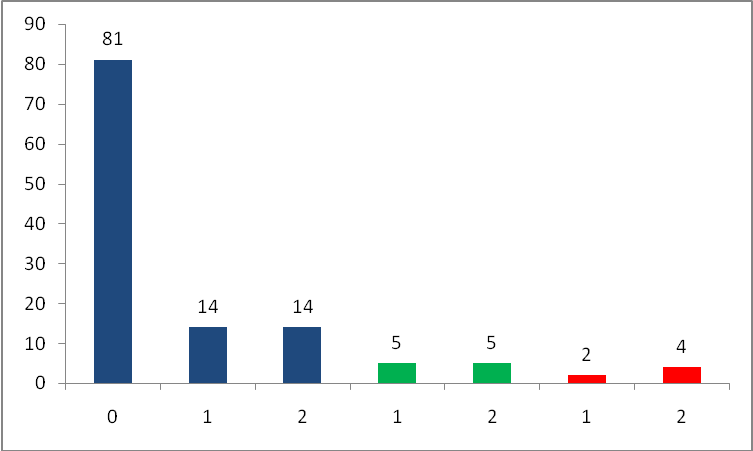


**Figure 4**. Distribution of phases among 125 pairs of overlapped genes in 6 *Escherichia coli* genomes and one plasmid. Blue, genes overlapped in different DNA strains (+/-) or (-/+); green and red, genes overlapped in the same DNA strain (+/+ and -/- respectively) but differing in 1 or 2 nucleotides in their frame shift. The 125 pairs of overlapped genes represent, each one, a separated family as defined by BLAST searches (e-value, 0.00001).


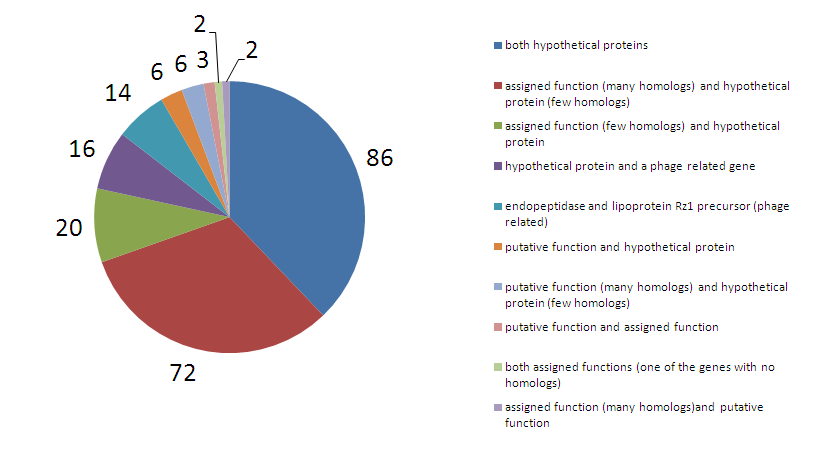


**Figure 5**. Classification of functions among 227 pairs of overlapped genes in 6 *Escherichia coli* genomes and one plasmid.


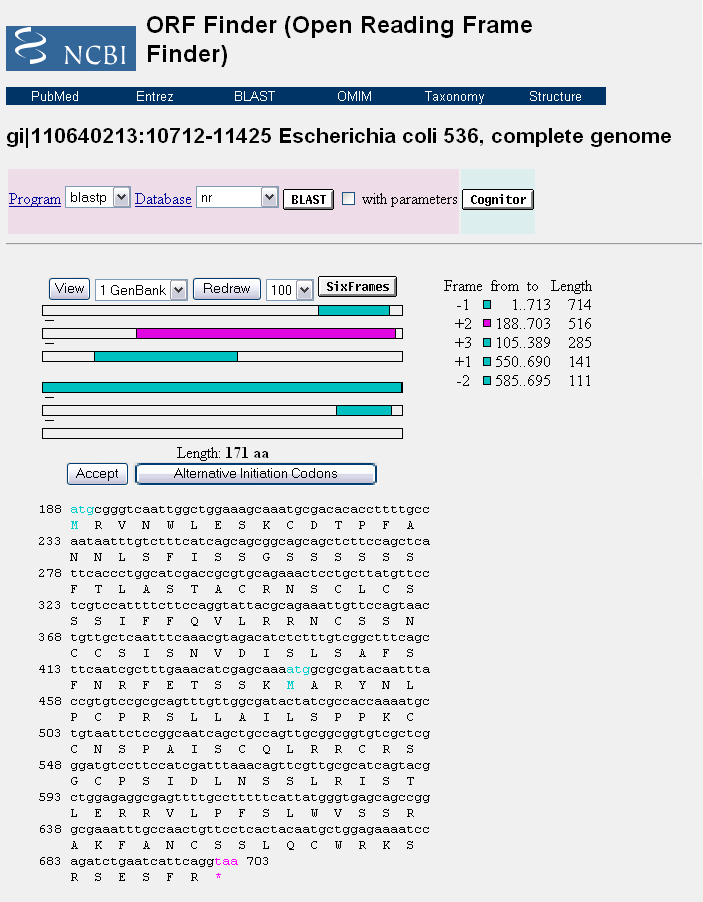


**Figure 6**. Putative overlapped *htgA* gene identified in *Escherichia coli* 536 (NC_008253) using ORF finder (www.ncbi.nlm.nih.gov/gorf/gorf.html).


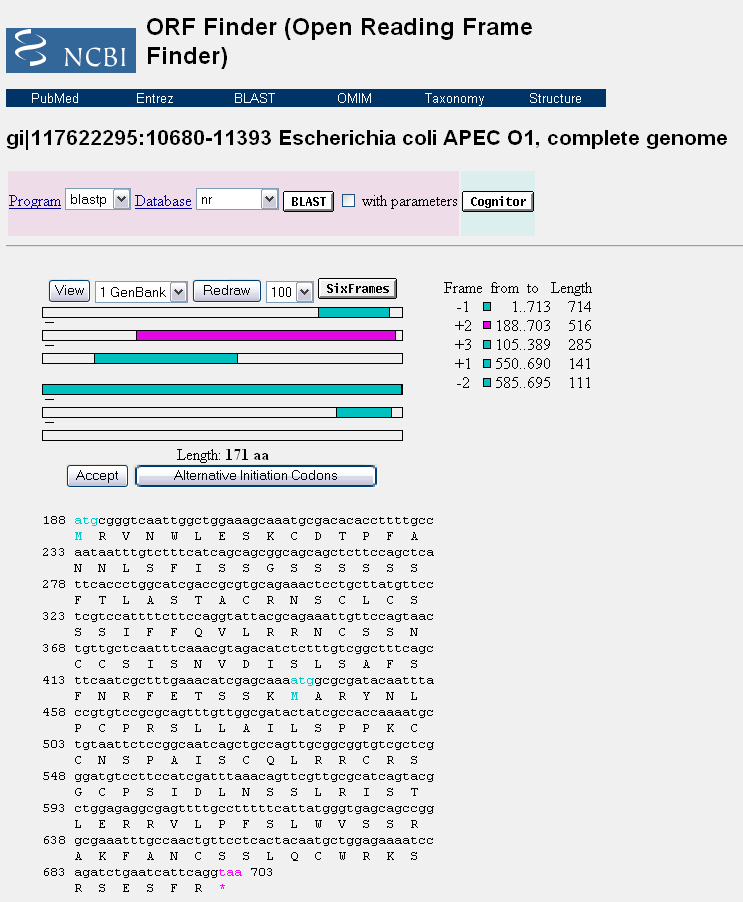


**Figure 7**. Putative overlapped *htgA* gene identified in *Escherichia coli* APEC O1 (NC_008563) using ORF finder (www.ncbi.nlm.nih.gov/gorf/gorf.html).

**Figure 8**. List of strict overlapped genes found in the eight *E. coli* genomes studied here. The list is divided according to functional categories.

******************************************************************************

******************************************************************************

Example:

89106895 29 hypothetical protein

^ ^ ^

| | |

| | |

gi|number Nunber of Function

homologs

******************************************************************************

******************************************************************************

Putative function and assigned function

(These pairs correspond to the htgA-yaaW genes studied in this analysis)

15799691 31 putative oxidoreductase

15799692 5 positive regulator for sigma 32 heat shock promoters

-----

15829265 31 putative oxidoreductase

15829266 5 positive regulator for sigma 32 heat shock promoters

-----

91209067 31 putative oxidoreductase

91209068 5 positive regulator for sigma 32 heat shock promoters

******************************************************************************

Assigned function (many homologs)and putative function

26245936 500 molecular chaperone DnaK

26245935 6 Putative glutamate dehydrogenase

-----

91209071 500 chaperone Hsp70; DNA biosynthesis; autoregulated heat shock proteins

91209070 6 putative glutamate dehydrogenase

-----

******************************************************************************

Both assigned functions (one of the genes with no homologs)

91211749 14 cell division protein ZipA

91211750 1 regulatory protein AlgP

-----

15800401 3 rhsC protein in rhs element, interrupted

15800402 1 rhsC protein in rhs element

-----

******************************************************************************

Both hypothetical proteins

89106895 29 hypothetical protein

89106896 5 hypothetical protein

-----

15803511 2 hypothetical protein Z4318

15803512 0 hypothetical protein Z4320

-----

15804119 500 hypothetical protein Z4998

15804120 1 hypothetical protein Z4999

-----

15830168 1 hypothetical protein ECs0914

38703896 15 hypothetical protein ECs5399

-----

15830830 1 hypothetical protein ECs1576

38703941 2 hypothetical protein ECs5420

-----

15831200 16 hypothetical protein ECs1946

15831201 1 hypothetical protein ECs1947

-----

38703970 7 hypothetical protein ECs5438

15831227 2 hypothetical protein ECs1973

-----

38703990 38 hypothetical protein ECs5444

15831396 1 hypothetical protein ECs2142

-----

38703994 7 hypothetical protein ECs5451

15831503 2 hypothetical protein ECs2249

-----

15833818 2 hypothetical protein ECs4564

38704191 1 hypothetical protein ECs5539

-----

38704248 15 hypothetical protein ECs5228

15834481 0 hypothetical protein ECs5227

-----

26246160 1 hypothetical protein c0253

26246161 25 hypothetical protein c0254

-----

26246166 124 hypothetical protein c0259

26246167 2 hypothetical protein c0260

-----

26246204 1 hypothetical protein c0299

26246205 6 hypothetical protein c0300

-----

26246256 1 hypothetical protein c0358

26246257 1 hypothetical protein c0359

-----

26246444 2 hypothetical protein c0549

26246445 5 hypothetical protein c0550

-----

26246485 2 hypothetical protein c0590

26246486 333 hypothetical protein c0591

-----

26246777 2 hypothetical protein c0887

26246778 50 Hypothetical protein ybiI

-----

26246939 12 Hypothetical protein ycaI

26246940 1 hypothetical protein c1053

-----

26247057 2 hypothetical protein c1172

26247058 45 hypothetical protein c1173

-----

26247063 10 hypothetical protein c1184

26247064 2 hypothetical protein c1185

-----

26247088 1 hypothetical protein c1209

26247089 7 hypothetical protein c1210

-----

26247165 28 hypothetical protein c1292

26247166 1 hypothetical protein c1293

-----

26247237 1 hypothetical protein c1367

26247238 2 hypothetical protein c1368

-----

26247290 8 hypothetical protein c1421

26247291 3 hypothetical protein c1422

-----

26247337 0 hypothetical protein c1468

26247338 0 hypothetical protein c1469

-----

26247350 1 hypothetical protein c1481

26247351 1 hypothetical protein c1482

-----

26247358 1 hypothetical protein c1489

26247359 1 hypothetical protein c1490

-----

26247459 5 hypothetical protein c1591

26247460 0 hypothetical protein c1592

-----

26247626 1 hypothetical protein c1762

26247627 1 hypothetical protein c1763

-----

26247674 5 hypothetical protein c1812

26247675 1 hypothetical protein c1813

-----

26247976 2 hypothetical protein c2122

26247977 20 Hypothetical protein ydiZ

-----

26248060 2 hypothetical protein c2206

26248061 152 hypothetical protein c2207

-----

26248313 3 hypothetical protein c2461

26248314 1 hypothetical protein c2462

-----

26248367 8 hypothetical protein c2520

26248368 1 hypothetical protein c2521

-----

26248412 2 hypothetical protein c2564

26248413 0 hypothetical protein c2565

-----

26248992 4 hypothetical protein c3150

26248993 2 hypothetical protein c3151

-----

26249056 2 hypothetical protein c3214

26249057 22 Hypothetical protein yqaE

-----

26249113 20 hypothetical protein c3274

26249114 1 hypothetical protein c3275

-----

26249144 2 hypothetical protein c3305

26249145 500 hypothetical protein c3306

-----

26249176 7 hypothetical protein c3338

26249177 2 hypothetical protein c3339

-----

26249178 7 hypothetical protein c3340

26249179 2 hypothetical protein c3341

-----

26249238 4 hypothetical protein c3403

26249239 2 hypothetical protein c3404

-----

26249414 1 hypothetical protein c3579

26249415 73 hypothetical protein c3580

-----

26249501 9 Hypothetical protein ykfF

26249502 2 hypothetical protein c3667

-----

26249507 1 hypothetical protein c3672

26249508 29 Hypothetical protein yeeT

-----

26249510 2 hypothetical protein c3675

26249511 44 Hypothetical protein yeeU

-----

26249660 3 hypothetical protein c3829

26249661 356 Hypothetical protein ygjH

-----

26249693 21 Hypothetical protein yhaK

26249694 1 hypothetical protein c3865

-----

26249728 29 hypothetical protein c3901

26249729 2 hypothetical protein c3902

-----

26250103 1 hypothetical protein c4281

26250104 4 hypothetical protein c4282

-----

26250181 2 hypothetical protein c4359

26250182 2 hypothetical protein c4360

-----

26250374 2 hypothetical protein c4554

26250375 0 hypothetical protein c4555

-----

26250378 6 hypothetical protein c4558

26250379 2 hypothetical protein c4559

-----

26250398 24 hypothetical protein c4578

26250399 1 hypothetical protein c4579

-----

26250584 22 Hypothetical protein yahG

26250585 1 hypothetical protein c4766

-----

26250972 2 hypothetical protein c5164

26250973 1 hypothetical protein c5165

-----

26250999 3 hypothetical protein c5191

26251000 21 hypothetical protein c5192

-----

26251007 3 hypothetical protein c5199

26251008 1 hypothetical protein c5200

-----

26251019 3 hypothetical protein c5211

26251020 1 hypothetical protein c5212

-----

26251182 1 hypothetical protein c5374

26251183 2 hypothetical protein c5375

-----

26251187 48 hypothetical protein c5379

26251188 1 hypothetical protein c5380

-----

91209454 2 hypothetical protein UTI89_C0408

91209455 20 hypothetical protein UTI89_C0409

-----

91209507 2 hypothetical protein UTI89_C0461

91209508 5 hypothetical protein UTI89_C0462

-----

91209544 2 hypothetical protein UTI89_C0498

91209545 333 hypothetical protein UTI89_C0499

-----

91209603 1 hypothetical protein UTI89_C0557

91209604 73 hypothetical protein UTI89_C0558

-----

91209830 1 hypothetical protein UTI89_C0799

91209831 2 hypothetical protein UTI89_C0800

-----

91210125 2 hypothetical protein UTI89_C1096

91210126 44 hypothetical protein UTI89_C1097

-----

91210133 10 hypothetical protein UTI89_C1104

91210134 2 hypothetical protein UTI89_C1105

-----

91210297 3 hypothetical protein UTI89_C1270

91210298 6 hypothetical protein UTI89_C1271

-----

91210335 2 hypothetical protein UTI89_C1308

91210336 3 hypothetical protein UTI89_C1309

-----

91210787 2 hypothetical protein UTI89_C1764

91210788 29 hypothetical protein UTI89_C1765

-----

91210938 2 hypothetical protein UTI89_C1917

91210939 20 hypothetical protein UTI89_C1918

-----

91211172 3 hypothetical protein UTI89_C2154

91211173 18 hypothetical protein UTI89_C2155

-----

91211248 2 hypothetical protein UTI89_C2235

91211249 2 hypothetical protein UTI89_C2236

-----

91211702 3 hypothetical protein UTI89_C2691

91211703 6 hypothetical protein UTI89_C2692

-----

91212022 2 hypothetical protein UTI89_C3021

91212023 22 hypothetical protein UTI89_C3022

-----

91212140 7 hypothetical protein UTI89_C3143

91212141 2 hypothetical protein UTI89_C3144

-----

91212142 7 hypothetical protein UTI89_C3145

91212143 2 hypothetical protein UTI89_C3146

-----

91212178 2 hypothetical protein UTI89_C3181

91212179 53 hypothetical protein UTI89_C3182

-----

91212204 4 hypothetical protein UTI89_C3210

91212205 2 hypothetical protein UTI89_C3211

-----

91212565 29 hypothetical protein YraN

91212566 2 hypothetical protein UTI89_C3575

-----

91212674 3 hypothetical protein UTI89_C3685

91212675 6 hypothetical protein UTI89_C3686

-----

91213929 6 hypothetical protein UTI89_C4981

91213930 2 hypothetical protein UTI89_C4982

-----

91213939 36 hypothetical protein UTI89_C4991

91213940 10 hypothetical protein UTI89_C4992

-----

91214080 4 hypothetical protein UTI89_C5135

91214081 2 hypothetical protein UTI89_C5136

-----

******************************************************************************

Endopeptidase and lipoprotein Rz1 precursor

89107421 38 predicted murein endopeptidase

89107422 12 predicted lipoprotein

-----

89108211 38 predicted defective peptidase

89108212 12 predicted lipoprotein

-----

16128539 38 DLP12 prophage; predicted murein endopeptidase

94541098 12 DLP12 prophage; predicted lipoprotein

-----

15830074 15 putative endopeptidase

15830075 3 putative lipoprotein Rz1 precursor

-----

15830346 42 putative endopeptidase

15830347 12 putative lipoprotein Rz1 protein precursor

-----

15830469 42 putative endopeptidase

15830470 12 putative lipoprotein Rz1 precursor

-----

15830788 50 putative endopeptidase

15830789 9 putative lipoprotein Rz1 precursor

-----

15831437 42 putative endopeptidase

15831438 12 putative lipoprotein Rz1 precursor

-----

15831992 42 putative endopeptidase

15831993 12 putative lipoprotein precursor

-----

15831040 42 endopeptidase

15831041 12 lipoprotein Rz1 precursor

-----

15831510 42 endopeptidase

15831511 12 lipoprotein Rz1 precursor

-----

91212231 56 hypothetical lipoprotein

91212230 1 hypothetical protein UTI89_C3236

-----

15830877 37 putative endopeptidase

15830878 12 lipoprotein Rz1 precursor

-----

15832219 42 putative endopeptidase

15832220 12 lipoprotein Rz1 precursor

-----

******************************************************************************

Hypotetical protein and a phage related gene

15801304 11 putative integrase of prophage CP-933C

15801305 2 unknown protein encoded by prophage CP-933C

-----

26247352 10 Putative integrase of prophage

26247353 2 Unknown protein encoded by prophage

-----

26247434 4 Partial tonB-like membrane protein encoded within prophage

26247435 2 hypothetical protein c1566

-----

26247582 0 TonB protein

26247583 0 hypothetical protein c1718

-----

91210333 4 TonB-like membrane protein encoded within prophage

91210334 2 hypothetical protein UTI89_C1307

-----

26246852 27 Putative phage tail protein

26246851 1 hypothetical protein c0961

-----

26247319 20 Putative capsid protein of prophage

26247318 4 hypothetical protein c1449

-----

26247327 30 Putative tail component of prophage

26247326 2 hypothetical protein c1457

-----

26247403 16 Putative exonuclease encoded by prophage

26247404 1 hypothetical protein c1535

-----

26247443 21 Putative capsid protein of prophage

26247442 4 hypothetical protein c1573

-----

26249000 76 Putative tail component of prophage

26248999 6 hypothetical protein c3157

-----

91210751 5 putative phage-related membrane protein

91210750 1 hypothetical protein UTI89_C1727

-----

15801378 11 putative membrane protein of prophage CP-933X

15801379 2 unknown protein encoded by prophage CP-933X

-----

15830794 8 putative Dnase

38703937 4 hypothetical protein ECs5417

-----

15831043 8 putative Dnase

38703954 4 hypothetical protein ECs5431

-----

15831222 8 putative DNase

38703969 4 hypothetical protein ECs5437

-----

******************************************************************************

Assigned function (few homologs) and hypothetical protein

-----

26249665 17 Evolved beta-galactosidase beta-subunit

26249666 1 hypothetical protein c3835

-----

26250096 28 nickel responsive regulator

26250097 1 hypothetical protein c4275

-----

26250159 10 2-dehydro-3-deoxygluconokinase

26250160 2 hypothetical protein c4338

-----

26250350 2 acetate/propionate kinase

26250351 1 hypothetical protein c4531

-----

26250462 21 PTS system, beta-glucoside-specific IIABC component

26250463 1 hypothetical protein c4645

-----

26250773 10 Zinc resistance-associated protein precursor

26250774 1 hypothetical protein c4960

-----

91206303 34 resolvase

91206304 1 hypothetical protein UTI89_P059

-----

91206383 10 DNA helicase I

91206384 1 hypothetical protein UTI89_P139

-----

91210440 38 cation transport regulator

91210439 2 hypothetical protein UTI89_C1412

-----

91210476 0 energy transducing membrane protein tonb

91210477 0 hypothetical protein UTI89_C1452

-----

91212150 18 suppressor of inhibitory function of ChpA, PemI-like, autoregulated

91212149 1 hypothetical protein UTI89_C3152

-----

91212449 24 protein YgiN

91212448 2 hypothetical protein UTI89_C3456

-----

91210542 11 putative tail fiber protein

91210543 2 hypothetical protein UTI89_C1520

-----

26249405 6 Hemolysin A

26249406 1 hypothetical protein c3571

-----

26249463 13 shiF protein

26249464 0 hypothetical protein c3629

-----

26248202 12 Protein ybcL precursor

26248203 1 hypothetical protein c2351

-----

26246719 20 Hypothetical protein ybgS precursor

26246718 1 hypothetical protein c0828

-----

26247895 19 Hypothetical protein ydhL precursor

26247896 1 hypothetical protein c2041

-----

26249215 53 Hypothetical lipoprotein ygdI precursor

26249214 2 hypothetical protein c3379

-----

26250806 0 Hypothetical protein yjbE precursor

26250805 1 hypothetical protein c4993

-----

******************************************************************************

Putative function and hypothetical protein

15802461 44 putative invasin

15802462 1 hypothetical protein Z3136

-----

26246224 16 Putative oligogalacturonide lyase

26246225 1 hypothetical protein c0320

-----

26246228 1 Putative exopolygalacturonate lyase

26246229 0 hypothetical protein c0324

-----

26251090 57 Unknown pentitol phosphotransferase enzyme II, B component

26251089 2 hypothetical protein c5282

-----

26250853 4 Putative transport sensor protein

26250854 1 hypothetical protein c5042

-----

15831885 3 putative derepression protein

15831884 1 hypothetical protein ECs2630

-----

******************************************************************************

Putative function (many homologs) and hypothetical protein (few homologs)

91211932 163 putative YhbH sigma 54 modulator

91211931 2 hypothetical protein UTI89_C2930

-----

91212164 215 probable serine transporter

91212163 2 hypothetical protein UTI89_C3166

-----

91212504 356 putative tRNA synthetase

91212503 3 hypothetical protein UTI89_C3512

-----

91212960 154 possible RNA-binding protein required for wild-type FtsZ ring formation on rich media

91212959 2 hypothetical protein UTI89_C3985

-----

26249478 126 Unknown in ISEc8

26249479 2 hypothetical protein c3644

-----

26249495 227 Unknown protein encoded by ISEc8

26249496 3 hypothetical protein c3661

-----

******************************************************************************

Assigned function (many homologs) and hypothetical protein (few homologs)

15803180 123 transport permease protein of gamma-aminobutyrate

15803181 1 hypothetical protein Z3962

-----

15832093 256 GDP-D-mannose dehydratase

38704044 1 hypothetical protein ECs5479

-----

15832356 209 O6-methylguanine-DNA methyltransferase

38704064 3 hypothetical protein ECs5484

-----

26246000 159 isopropylmalate isomerase small subunit

26246001 1 hypothetical protein c0088

-----

26246368 500 Taurine transport ATP-binding protein tauB

26246369 1 hypothetical protein c0474

-----

26246422 394 transcription antitermination protein NusB

26246421 3 hypothetical protein c0526

-----

26246463 500 Nitrogen Regulatory protein P-II 2

26246464 2 hypothetical protein c0569

-----

26246535 500 Peptidyl-prolyl cis-trans isomerase B

26246534 3 hypothetical protein c0640

-----

26246598 357 Citrate lyase beta chain

26246597 2 hypothetical protein c0705

-----

26246688 100 succinate dehydrogenase cytochrome b556 large membrane subunit

26246689 1 hypothetical protein c0799

-----

26246711 500 Peptidoglycan-associated lipoprotein precursor

26246710 2 hypothetical protein c0820

-----

26246741 61 Peyer's patch-specific virulence factor GipA

26246742 1 hypothetical protein c0852

-----

26246743 143 adenosylmethionine--8-amino-7-oxononanoate transaminase

26246744 1 hypothetical protein c0854

-----

26246842 45 Major capsid protein

26246841 1 hypothetical protein c0951

-----

26246958 104 nicotinate phosphoribosyltransferase

26246959 1 hypothetical protein c1074

-----

26247234 403 acyl carrier protein

26247233 3 hypothetical protein c1363

-----

26247539 463 2-dehydro-3-deoxyphosphooctonate aldolase

26247538 1 hypothetical protein c1673

-----

26247763 500 Formate dehydrogenase-N beta subunit

26247762 3 hypothetical protein c1905

-----

26248085 500 Cold shock-like protein cspC

26248084 7 hypothetical protein c2230

-----

26248158 71 Flagellar transcriptional activator flhC

26248159 1 hypothetical protein c2307

-----

26248219 179 flagellar biosynthesis protein

26248220 1 hypothetical protein c2368

-----

26248235 112 Outer membrane protein N precursor

26248236 1 hypothetical protein c2384

-----

26248352 102 Transposase

26248353 1 hypothetical protein c2504

-----

26248523 21 cytidine deaminase

26248524 1 hypothetical protein c2676

-----

26248600 208 ADA Regulatory protein

26248601 3 hypothetical protein c2755

-----

26248686 500 phosphate acetyltransferase

26248685 3 hypothetical protein c2839

-----

26248883 478 nucleoside diphosphate kinase

26248882 2 hypothetical protein c3040

-----

26248897 375 NifU-like protein

26248896 1 hypothetical protein c3054

-----

26248960 163 Protein yfiA

26248959 2 hypothetical protein c3118

-----

26248977 466 GrpE protein

26248976 4 hypothetical protein c3134

-----

26249201 215 Serine transporter

26249200 2 hypothetical protein c3363

-----

26249226 123 Secreted protein Hcp

26249225 6 hypothetical protein c3390

-----

26249652 500 RpoD protein

26249651 3 hypothetical protein c3820

-----

26249768 250 50S ribosomal protein L27

26249769 0 hypothetical protein c3943

-----

26249889 500 30S ribosomal protein S5

26249888 3 hypothetical protein c4064

-----

26249894 500 30S ribosomal protein S8

26249893 2 hypothetical protein c4069

-----

26249899 500 50S ribosomal protein L14

26249898 2 hypothetical protein c4074

-----

26249906 493 50S ribosomal protein L22

26249905 3 hypothetical protein c4081

-----

26249909 500 50S ribosomal protein L2

26249908 2 hypothetical protein c4084

-----

26249911 329 50S ribosomal protein L23

26249910 1 hypothetical protein c4086

-----

26250199 500 Cold shock protein cspA

26250198 4 hypothetical protein c4376

-----

26250475 385 ATP synthase subunit epsilon

26250474 3 hypothetical protein c4656

-----

26250592 360 Uridine phosphorylase

26250591 1 hypothetical protein c4772

-----

26250753 500 50S ribosomal protein L11

26250752 3 hypothetical protein c4938

-----

26250757 475 50S ribosomal protein L7/L12

26250756 3 hypothetical protein c4942

-----

26250894 500 Formate dehydrogenase H

26250895 1 hypothetical protein c5083

-----

26251034 500 co-chaperonin GroES

26251033 1 hypothetical protein c5225

-----

26251101 464 50S ribosomal protein L9

26251100 1 hypothetical protein c5293

-----

91209485 394 FJECB transcription termination; L factor

91209484 3 hypothetical protein UTI89_C0438

-----

91209498 148 cytochrome O ubiquinol oxidase protein CyoD

91209499 1 hypothetical protein UTI89_C0453

-----

91209665 357 citrate lyase beta chain (acyl lyase subunit)

91209664 2 hypothetical protein UTI89_C0619

-----

91209767 446 cytochrome d terminal oxidase polypeptide subunit II

91209766 3 hypothetical protein UTI89_C0728

-----

91209776 500 peptidoglycan-associated lipoprotein precursor

91209775 2 hypothetical protein UTI89_C0737

-----

91210247 403 acyl carrier protein

91210246 3 hypothetical protein UTI89_C1219

-----

91210536 76 minor tail protein

91210535 6 hypothetical protein UTI89_C1512

-----

91210714 500 formate dehydrogenase-N beta subunit

91210713 3 hypothetical protein UTI89_C1690

-----

91211505 209 Ada transcriptional dual regulator / O-6-methylguanine-DNA methyltransferase

91211506 3 hypothetical protein UTI89_C2494

-----

91211593 500 phosphate acetyltransferase

91211592 3 hypothetical protein UTI89_C2580

-----

91211845 478 nucleoside diphosphate kinase

91211844 2 hypothetical protein UTI89_C2839

-----

91211948 467 GrpE protein

91211947 4 hypothetical protein UTI89_C2946

-----

91212190 122 Secreted protein Hcp

91212189 6 hypothetical protein UTI89_C3195

-----

91212609 474 50S ribosomal subunit protein L21

91212608 4 hypothetical protein UTI89_C3619

-----

91212732 500 30S ribosomal subunit protein S5

91212731 3 hypothetical protein UTI89_C3749

-----

91212737 500 30S ribosomal subunit protein S8, and regulator

91212736 2 hypothetical protein UTI89_C3754

-----

91212742 500 50S ribosomal subunit protein L14

91212741 2 hypothetical protein UTI89_C3759

-----

91212749 493 50S ribosomal subunit protein L22

91212748 3 hypothetical protein UTI89_C3766

-----

91212752 500 50S ribosomal subunit protein L2

91212751 2 hypothetical protein UTI89_C3769

-----

91212808 475 50S ribosomal subunit protein L7/L12

91212807 3 hypothetical protein UTI89_C3833

-----

91212812 500 50S ribosomal subunit protein L11

91212811 3 hypothetical protein UTI89_C3837

-----

91213070 500 cold shock protein 7.4, transcriptional activator of hns

91213069 4 hypothetical protein UTI89_C4096

-----

91213153 195 50S ribosomal subunit protein L33

91213152 1 hypothetical protein UTI89_C4179

-----

91213256 385 membrane-bound ATP synthase F1 sector epsilon-subunit

91213255 3 hypothetical protein UTI89_C4283

-----

******************************************************************************
